# Nitrogen-Doped Carbon for Red Phosphorous Based Anode Materials for Lithium Ion Batteries

**DOI:** 10.3390/ma11010134

**Published:** 2018-01-15

**Authors:** Jiaoyang Li, Yumin Qian, Li Wang, Xiangming He

**Affiliations:** 1 Institute of Nuclear & New Energy Technology, Tsinghua University, Beijing 100084, China; ljyljy1801@126.com; 2 Department of Mechanical Engineering, Massachusetts Institute of Technology, Cambridge, MA 02139, USA; 3 Institute of Functional Nano & Soft Materials, Soochow University, Suzhou 215123, China; yuminqianwk@foxmail.com

**Keywords:** nitrogen-doped carbon, red phosphorous, anode materials, lithium ion batteries

## Abstract

Serving as conductive matrix and stress buffer, the carbon matrix plays a pivotal role in enabling red phosphorus to be a promising anode material for high capacity lithium ion batteries and sodium ion batteries. In this paper, nitrogen-doping is proved to effective enhance the interface interaction between carbon and red phosphorus. In detail, the adsorption energy between phosphorus atoms and oxygen-containing functional groups on the carbon is significantly reduced by nitrogen doping, as verified by X-ray photoelectron spectroscopy. The adsorption mechanisms are further revealed on the basis of DFT (the first density functional theory) calculations. The RPNC (red phosphorus/nitrogen-doped carbon composite) material shows higher cycling stability and higher capacity than that of RPC (red phosphorus/carbon composite) anode. After 100 cycles, the RPNC still keeps discharge capacity of 1453 mAh g^−1^ at the current density of 300 mA g^−1^ (the discharge capacity of RPC after 100 cycles is 1348 mAh g^−1^). Even at 1200 mA g^−1^, the RPNC composite still delivers a capacity of 1178 mAh g^−1^. This work provides insight information about the interface interactions between composite materials, as well as new technology develops high performance phosphorus based anode materials.

## 1. Introduction

Lithium ion batteries (LIBs) are expected to be the prime candidates for energy storage and power application, owing to their high energy efficiency, high energy density, and long cycle lifetime [1,2,3]. The ever increasing application of LIBs in electric vehicles and portable devices has promoted the urgent demand for LIBs with improved energy density [4,5,6]. However, the conventional graphite anode can hardly meet the demands with a low theoretical capacity of 372 mAh g^−1^ [7,8]. Therefore, new anode materials with high capacity have attracted increasing attention. Red phosphorus is a promising next-generation anode material for lithium ion batteries, due to extremely high theoretical specific capacity (2596 mAh g^−1^), low-cost, and abundant available [9,10]. However, the practical application of red phosphorus is hindered by several issues [11,12]. First, the phosphorus inherent low electrical conductivity that necessitates the use of conductive carbon to improve its utilization. Second, the volumetric expansion of phosphorus (around 270%) leads to poor cycling stability. Thus a variety of strategies have been explored to achieve advanced phosphorous-based electrode. Among of them, compositing with carbon is proved to be effective in enabling red phosphorus to deliver high capacity and cycleability despite of the poor electrical conductivity and dramatic volume change during lithiation/delithiation of the red phosphorus. Acting as both a conductive matrix and nano-particle carrier, the conductivity, surface affinity to electrolyte and phosphorus are determining properties for the composite performances. Therefore, the development of novel functionalized carbon with the ability to adsorb phosphorus is particularly attractive. Nitrogen-doping is proved to facilitate lithiation, due to its higher electronegativity [13,14]. Nitrogen-doping into carbon materials effective enhance electronic conductivity, and moreover, its heteropolarity make it an active site for bonding or absorption for lithium ions, polysulfides, and oxides [15,16].

In this work, the function of nitrogen-doping on phosphorus absorption or bonding is investigated. A simple and fast solvothermal was employed to prepared nitrogen-doped porous carbon (NC), which is then used as the conductive matrix to prepare a red phosphorus/nitrogen-doped carbon composite (RPNC). The investigations on its lithium storage performance demonstrate that nitrogen-doping enables the porous carbon higher ability to hold phosphorus particles, and so improve the electrochemical performances of phosphorus-based electrode in terms of capacity and cycleability.

## 2. Experimental

Materials: 1 g commercial active carbon was dispersed in 60 mL mixed solvent of ethyl alcohol and deionized water (1:1 *v*/*v*) contained 20 wt % NH_4_Cl under continuous stirring at room temperature. After being stirred, the solution was transferred into Teflon-lined stainless steel autoclave. Then, the autoclave was heated and the temperature was maintained at 120 °C for 5 h. After being cooled to room temperature, the product was collected by centrifugation and washed several times with deionized water, followed by drying in a vacuum oven at 80 °C for 12 h. The dried product was then annealed in a tube furnace at 450 °C for 3 h in a nitrogen atmosphere, with a ramp rate of 3 °C min^−1^, to obtain the nitrogen doped carbon.

The rep phosphorus/nitrogen doped carbon (RPNC) composite was prepared by evaporation-adsorption process. The red phosphorus and nitrogen doped carbon were separately placed in sealed steel vessel, which was filled with pure argon. Then, the vessel was heated at 450 °C for 3 h and the RPNC composite was obtained after cooling to room temperature. The rep phosphorus/carbon (RPC) composite was prepared in the same way.

Characterization: The structure was characterized by X-ray diffraction (XRD, Rigaku D/max-2500) (Akishima, Japan) with Cu-Kα radiation. The surface morphology of the composite was examined by scanning electron microscopy (SEM, JEOL-6301F) (Tokyo, Japan) with energy dispersive spectroscopy (EDS) mapping. X-ray photoelectron spectroscopy (XPS) measurements were carried out on Axis Ultra of Kratos Analytical Ltd. (Manchester, UK), all of the calculations were performed by the first principles density functional theory implemented with Gaussian 09 Software package.

Electrochemical characterization: As-prepared composite (RPNC and RPC), acetylene black and polyacrylonitrile (with a weight ratio of 70:20:10), were homogeneously mixed and made a thin film as the working electrode. Then, the electrodes were dried in a vacuum oven at 80 °C for 12 h. Finally, CR2032coin-type cells were assembled in an argon-filled glove box, with the composite as cathode, metallic lithium disk as anode, Celgard2400 as separator. 1 M LiPF_6_ dissolved in a mixed solvent of ethylene carbonate (EC), dimethyl carbonate (DMC), and ethyl methyl carbonate (EMC) (1:1:1 by volume.) was used as electrolyte in this work. Galvanostatic charge-discharge cycles with a current density of 300 mA g^−1^ between 0.001 and 2 V were performed using Land CT2001A (Wuhan, China). Cyclic voltammetry (CV) measurements were conducted on CHI 660E (Shanghai, China) within a voltage range of 0.001–2 V at a scan rate of 0.2 mV s^−1^. The Electrochemical Impedance Spectra (EIS) were also performed on CHI 660E.

## 3. Results and Discussion

**Figure 1:**
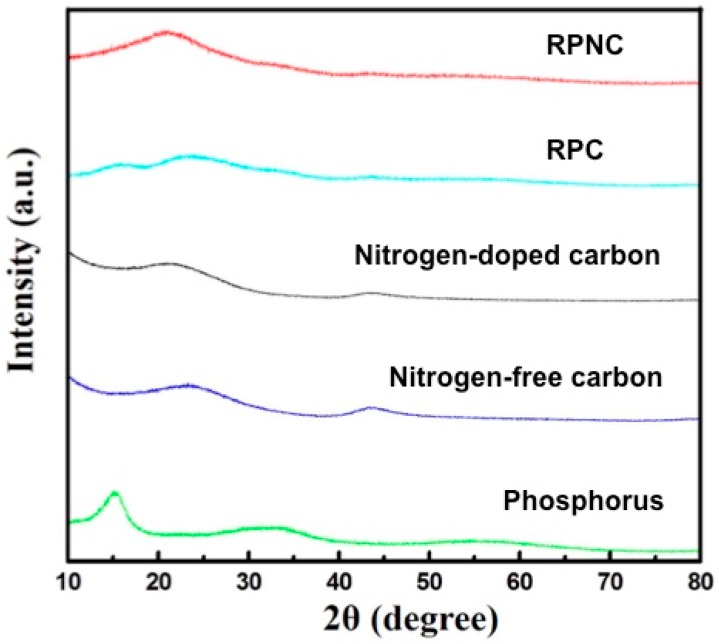
XRD patterns of elemental phosphorus, nitrogen-free carbon, nitrogen-doped carbon, and composites.

Figure 1 shows the XRD patterns of elemental phosphorus, nitrogen-free carbon, NC, and their composites (RPC and RPNC). In XRD pattern of phosphorus, a typical characteristic peaks of phosphorus appears around 2θ = 15°, corresponding to (003) diffraction modes (JCPDS No. 44-0906) [17,18]. Nitrogen-doped carbon exhibits two broad bands around 2θ = 22° and 43°, respectively [19,20]. As NC has similar diffraction peak with the pristine carbon (Figure S1), it can be concluded that nitrogen-doping does not cause observable variation in the crystal structure of carbon. Interestingly, different with red phosphorus/carbon (RPC), RPNC does not show the characteristic peak of phosphorus (~15°) in the XRD pattern. This observation suggests that phosphorus particles absorbed and precipitated is crystal in pristine porous carbon, while is amorphous when the surface of the carbon is doped with nitrogen. Therefore, the differences between RPNC and RPC are from nitrogen–doping, rather than the crystal structure change before and after nitrogen–doped.

**Figure 2:**
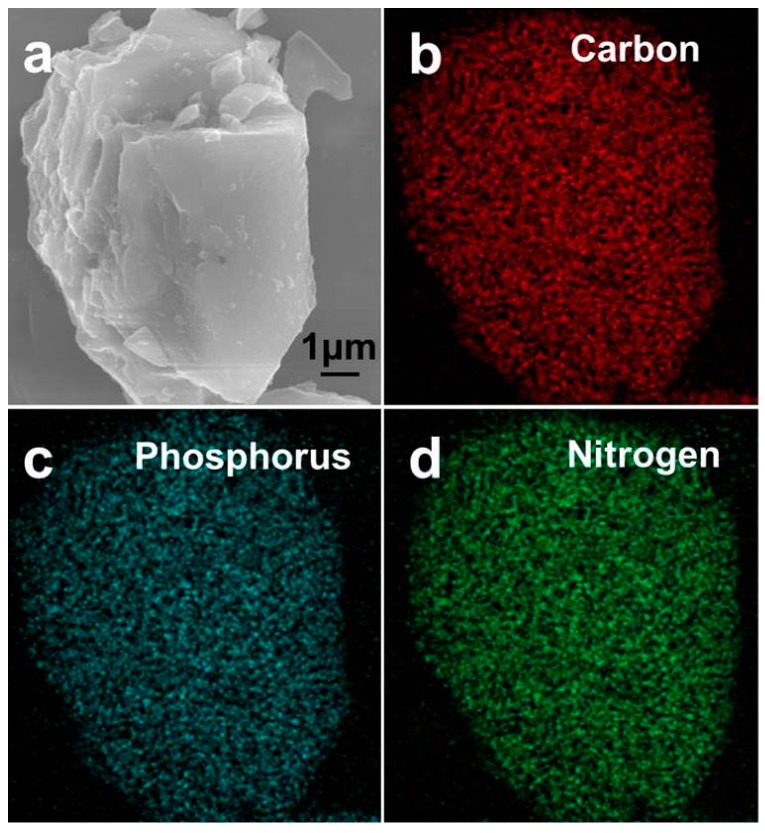
(**a**) SEM images of a red phosphorus/nitrogen-doped carbon composite (RPNC) composite particle; (**b**–**d**) Energy Dispersive Spectrometer (EDS) mapping showing distribution of carbon, phosphorus and nitrogen.

The surface morphology of the RPNC is shown in the SEM images (Figure 2a). It can be seen that the composite has layer structures with smooth surface. The layer structures not only contribute to loading phosphorus, but also benefit for electrolytes access, facilitating the lithiation/delithiation. Figure 2b–d show the EDS (Energy Dispersive Spectrometer) elemental mapping images of the RPNC composites. The elemental mapping image of carbon (Figure 2b) is almost identical with that of phosphorus (Figure 2c), indicating the uniform distribution of phosphorus in composites. The existence of nitrogen in RPNC is confirmed in Figure 2d, while it cannot be detected in RPC (Figure S1, Supporting Information), verifying that the nitrogen in RPNC comes from doping treatment instead of the native composition of the carbon. In addition, the elemental analysis shows that the nitrogen content in nitrogen–doped carbon is up to 7.67%, as shown in Table 1. The result also is demonstrated by following XPS analysis.

**Figure 3:**
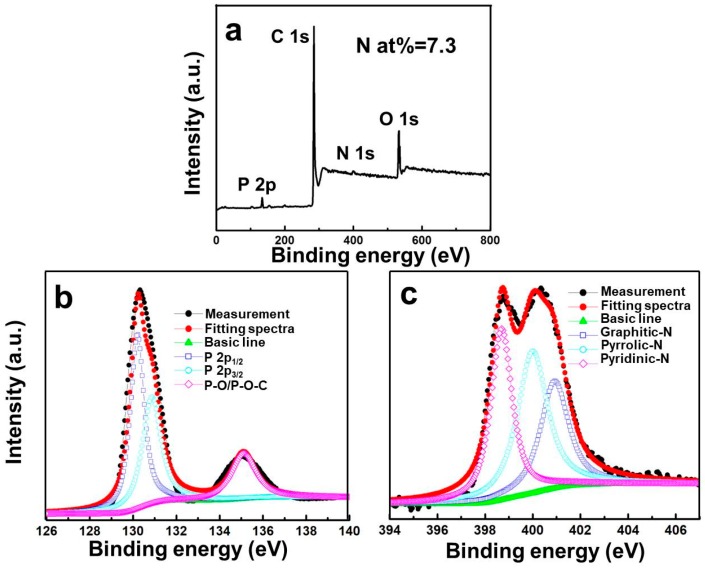
(**a**) X-ray photoelectron spectroscopy (XPS) survey spectra of RPNC; high-resolution XPS spectra of (**b**) P 2p and (**c**) N 1s in the RPNC.

The chemical composition and surface properties of the RPNC composite are analyzed further by XPS characterizations. The survey spectra in Figure 3a shows four peaks at 130, 290, 400, and 530 eV, which are attribute to P2p, C1s, N1s, and O1s, respectively. According to the fitting curve based on Gaussian function, the P2p spectra (Figure 3b) shows two peaks at around 129.9 eV and 130.7 eV, assignable to 2p_3/2_ and 2p_1/2_ binding energy, respectively [21]. A peak at high energy region (135.1 eV) is in indicative of the interaction between phosphorus and carbon [11,22]. The existence of P–O/P–O–C bonds enables the phosphorus to bind strongly with carbon, thereby contributing to ensure a strong and stable electrical contact between phosphorus and carbon matrix during electrochemical cycling [22]. Fitting with the Gaussian function, the N1s peak (Figure 3c) can be divided into pyridinic–N (398.7 eV), pyrrolic–N (400.3 eV), and graphitic–N (401.1 eV), respectively [23,24,25]. The total content of nitrogen in RPNC is up to 7.3 at %, which are matched with elemental analysis result mentioned above.

**Figure 4:**
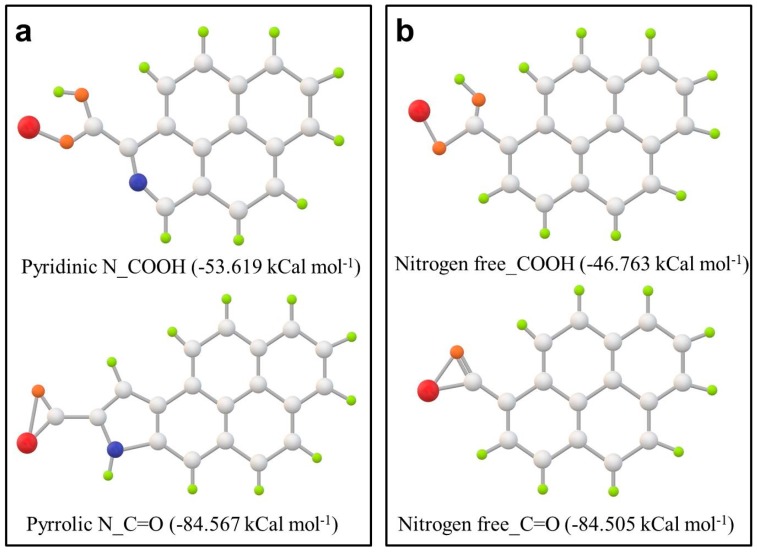
The optimized geometries of phosphorus adsorbed on oxygen-containing functional groups in both (**a**) NC and (**b**) pristine carbon, on the basis of first principles density functional theory (DFT) calculation result. Silver, red, blue, brown, and green balls represent carbon, phosphorus, nitrogen, oxygen, and hydrogen atoms, respectively.

DFT (first principles density functional theory) calculations are carried out to better understand bonding between nitrogen and phosphorus. The possible structures of phosphorus adsorbed on carboxyl or carbonyl groups in pyridinic–N–doped and pyrrolic–N–doped carbon are proposed in Figure 4 and Figure S2. Their interaction energies (*ΔE*) are calculated and the smaller value of *ΔE* is the more thermodynamically favorable of the adsorption mode well. As can be seen in Table 2, among three carboxyl groups on both the nitrogen–doped and nitrogen-free carbon, the *ΔE* of carboxyl group on pyridinic–N–doped carbon (−53.619 kCal mol^−1^) is the smallest, while the *ΔE* of the carbonyl group on pyrrolic–N–doped carbon (−84.567 kCal mol^−1^) is the smallest in all of the carboxyl groups. The lower *ΔE* of phosphorus adsorption on the oxygen-containing functional groups of the NC than that on corresponding pristine carbon indicates that N–doping facilitate phosphorus adsorption on the carbon matrix. It also can be observed that the interaction energy between phosphorus and nitrogen on both pyridinic N_COOH and pyrrolic N_C=O groups are somewhat weaker than that for all of the functional groups, revealing that the adsorption of phosphorus tends to happen on oxygen. The result is consistent with the above XPS results, which confirms the formation of P–O/P–O–C bond instead of P–N bond.

**Figure 5:**
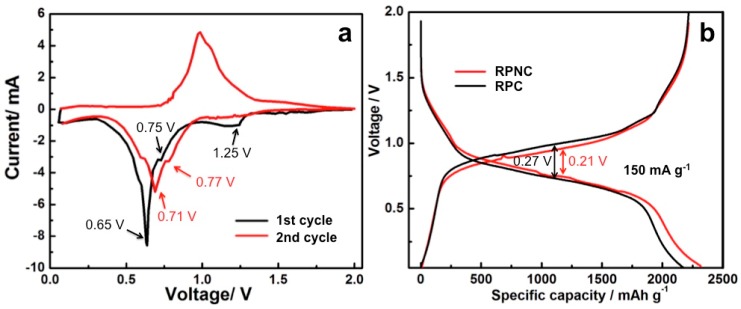
(**a**) The initial two cyclic voltammetry curves of RPNC composite at sweep speed of 0.2 mV s^−1^; (**b**) the second charge-discharge profiles of RPNC and RPC at the current density of 150 mA g^−1^.

The initial two cyclic voltammetry (CV) curves of a half cell with the RPNC composite are shown in Figure 5a. The initial discharge curve shows three reduction peaks at 1.25 V, 0.75 V, and 0.65 V, two reduction peaks at 0.71 V and 0.77 V appear in the second discharge curve. The disappeared peak at 1.25 V may be due to reduction of unknown species on the surface of the composites. The reduction peaks at 0.65–0.8 V agree well with the reported literatures and could be reasonably attributed to the formation of Li_x_P phases (*x* = 1–3) [26,27]. The initial charge data evidence two oxidation peaks at 1.0 V and 1.02 V, respectively, corresponding to the step-by-step delithiation of Li_3_P [26]. Figure 5b shows the comparison of the second charge-discharge profiles of RPNC and RPC. Both RPNC and RPC have somewhat inclined platforms locating at 0.75 V during lithiation and 1.0 V during delithiation, which correspond well with the transition between P and Li_3_P. When compared with RPC, RPNC has a prolonged plateau around 0.75 V, where there is an extra 170 mAh g^−1^ than RPC, and its reversible specific capacity is up to 2325 mAh g^−1^. Additionally, the prolonged plateau around 0.75 V reflects better kinetics of RPNC. More importantly, the voltage gap between lithiation and delithiation of RPNC (0.21 V) is lesser than NPC (0.27 V), which indicates the reduction of lithiation/delithiation polarization of RPNC. The polarization decrease achieved by the introduction of nitrogen, which could be very beneficial for utilization of low conductive phosphorus in the composite. The improvements in kinetics and polarization contribute to enhance the energy and power density.

**Figure 6:**
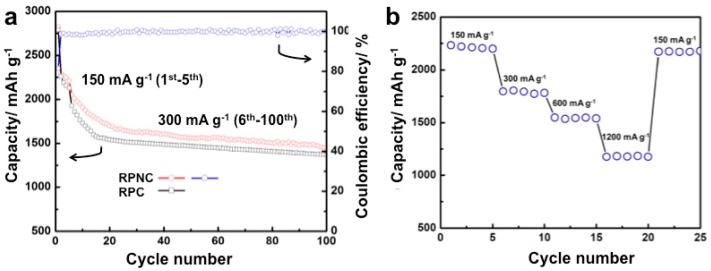
(**a**) The comparison of cycling performances and coulombic efficiency of RPNC and RPC; (**b**) the rate capability of RPNC composite at various current densities.

Figure 6a depicts the cycling performances of RPNC and RPC. The RPNC composite exhibits the initial lithiation capacity of 2822 mAh g^−1^, and decrease to 2210 mAh g^−1^ after five cycles at the current density of 150 mA g^−1^. Then, the discharge capacity gradually reduces to 1699 mAh g^−1^ from 2048 mAh g^−1^ (the 6th cycle) in the following fifteen cycles at the current density of 300 mA g^−1^. After 100 cycles, the discharge capacity of RPNC still keeps at 1453 mAh g^−1^ (2.0 mAh cm^−2^), which retains 71% of initial reversible discharge capacity at 300 mA g^−1^. Notably, the coulombic efficiency maintains above 98% from 2nd cycle to 100th cycle. As shown in Figure 6a, both the initial discharge capacity and cycling stability of RPNC is superior to that of RPC. The nitrogen-doped carbon is beneficial for adsorbing phosphorus in the physical and/or chemical way, thereby comprehensively enhancing the electrochemical properties. The rate performance of RPNC is also studied. Figure 6b displays the lithiation capacities of RPNC at various lithiation and delithiation rates between 150 mA g^−1^ and 1200 mA g^−1^ each sustained for 5 cycles. The RPNC delivers capacity of 2210 mAh g^−1^ at 150 mA g^−1^. Even at high current density of 1200 mA g^−1^, the lithiation capacity of RPNC composite is still as high as 1178 mAh g^−1^, exhibiting an excellent rate capacity. It should be noted that the reversible capacity is mostly recovered (2210 mAh g^−1^) when the discharge current density is turned back to 150 mA g^−1^. The good rate capability of RPNC can be attributed to excellent electrical conductivity and outstanding ability to accommodate volume expansion, which may benefit from nitrogen–doping [28,29]. Furthermore, the effect of N–doping on transmission of lithium ion and electron in RPNC composite is further demonstrated by the following Electrochemical Impedance Spectroscopy (EIS) measurements.

**Figure 7:**
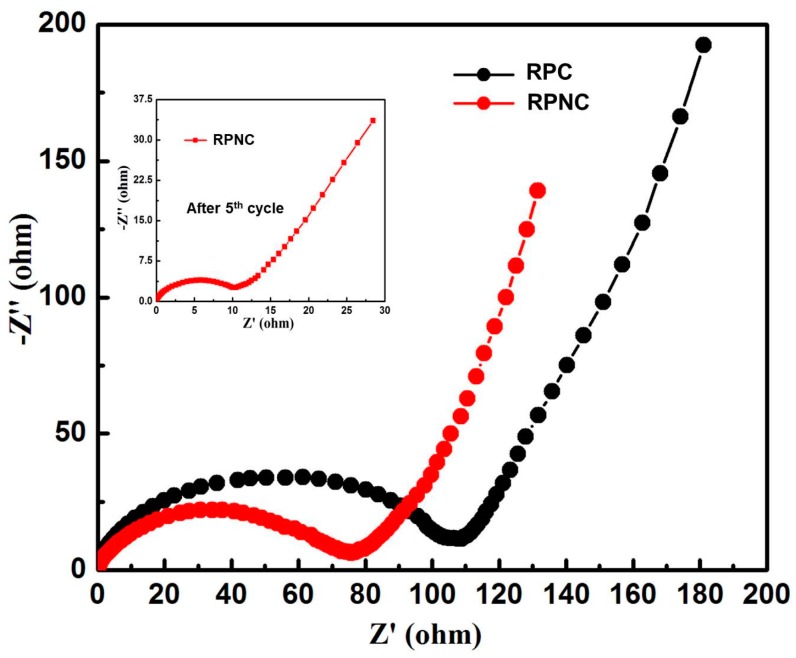
EIS analysis of the RPNC and rep phosphorus/carbon (RPC) composites after the initial charge/discharge cycle. The inset image is the EIS of RPNC after five cycles.

The EIS curves of RPNC and RPC are shown in Figure 7. It can be seen that the Nyquist plots of both composites represent a depressed semicircle in the high-to-medium frequency range, corresponding to the charge transfer impedance (R_ct_) at the electrode/electrolyte interface, followed by a straight line in low frequency, which stands for Warburg diffusion impedance and relates with the bulk diffusion resistance in composites [30,31,32]. It can be seen that the R_ct_ value of RPNC and RPC are 76 Ω and 108 Ω, respectively. The much lower R_ct_ value of RPNC indicates it has better electronic conductivity [33]. The improved conductivity is probably related to N–doping, which can facilitate the electron transport and improve the electrode conductivity. The straight slope of RPNC low frequency in low frequency is slightly higher than that of RPC, indicating better lithium ion diffusion ability in the NC matrix. The low charge transfer resistance value and the high lithium ion diffusion ability represent the excellent electronic conductivity and high lithium ion transfer speed across the interfaces between the electrolyte and the active electrode materials, which can enhance the lithiation/delithiation performance and rate capability of batteries. Notably, the impendence of RPNC decreases to 10 Ω after 5 cycles, maybe because the active phosphorus takes up more electrochemical favorable positions [32]. In addition, there is no clear change in the tracing pattern before and after cycling, implying formation of a stable and highly conductive solid electrolyte interphase, which is beneficial for improving the electrochemical performances.

## 4. Conclusions

In summary, nitrogen-doped carbon matrix is prepared by a simple solvothermal method, followed by thermal treatment under nitrogen atmosphere, and is used to fabricate red phosphorus based composites as high capacity anode materials for lithium ion batteries. The electronic conductivity and ion diffusion in RPNC are better than RPC. DFT calculations help understand the phosphorus adsorbing sites and interaction energies in NC. The calculations are further confirmed by XPS characterization. RPNC delivers reversible capacity of 1453 mAh g^−1^ after 100 cycles at a current density of 300 mA g^−1^ and capacity density of 2.0 mAh cm^−2^. Even at 1200 mA g^−1^, RPNC still delivers a capacity of 1178 mAh g^−1^ (1.6 mAh cm^−2^). All of the results show nitrogen doped carbon matrix effectively enhance the performances and utilization of phosphorus, making red phosphorus/nitrogen-doped carbon composite one of the promising candidates for anode materials of lithium-ion batteries.

## 5. Experimental

Materials: 1 g commercial active carbon was dispersed in 60 mL mixed solvent of ethyl alcohol and deionized water (1:1 *v*/*v*) contained 20 wt % NH_4_Cl under continuous stirring at room temperature. After being stirred, the solution was transferred into Teflon-lined stainless steel autoclave. Then the autoclave was heated and the temperature was maintained at 120 °C for 5 h. After being cooled to room temperature, the product was collected by centrifugation and washed several times with deionized water, followed by drying in a vacuum oven at 80 °C for 12 h. The dried product was then annealed in a tube furnace at 450 °C for 3 h in a nitrogen atmosphere, with a ramp rate of 3 °C min^−1^, to obtain the nitrogen doped carbon.

The rep phosphorus/nitrogen doped carbon (RPNC) composite was prepared by evaporation-adsorption process. The red phosphorus and nitrogen doped carbon were separately placed in sealed steel vessel, which was filled with pure argon. Then, the vessel was heated at 450 °C for 3 h and the RPNC composite was obtained after cooling to room temperature. The rep phosphorus/carbon (RPC) composite was prepared in the same way.

Characterization: The structure was characterized by X-ray diffraction (XRD, Rigaku D/max-2500) with Cu-Kα radiation. The surface morphology of the composite were examined by scanning electron microscopy (SEM, JEOL-6301F) with energy dispersive spectroscopy (EDS) mapping. X-ray photoelectron spectroscopy (XPS) measurements were carried out on Axis Ultra of Kratos Analytical Ltd. All of the calculations were performed by the first principles density functional theory implemented with Gaussian 09 software package.

Electrochemical characterization: As-prepared composite (RPNC and RPC), acetylene black and polyacrylonitrile (with a weight ratio of 70:20:10), were homogeneously mixed and made a thin film as the working electrode. Then, the electrodes were dried in a vacuum oven at 80 °C for 12 h. Finally, CR2032coin-type cells were assembled in an argon-filled glove box, with the composite as cathode, metallic lithium disk as anode, Celgard2400 as separator. 1 M LiPF_6_ dissolved in a mixed solvent of ethylene carbonate (EC), dimethyl carbonate (DMC), and ethyl methyl carbonate (EMC) (1:1:1 by volume.) was used as electrolyte in this work. Galvanostatic charge-discharge cycles with a current density of 300 mA g^−1^ between 0.001 and 2 V were performed using Land CT2001A. Cyclic voltammetry (CV) measurements were conducted on CHI 660E within a voltage range of 0.001–2 V at a scan rate of 0.2 mV s^−1^. The Electrochemical Impedance Spectra (EIS) were also performed on CHI 660E.

## Figures and Tables

**Table 1:** The carbon, oxygen and nitrogen atomic contents (%) of nitrogen-doped carbon and nitrogen-free carbon determined by elemental analysis.

Sample		Atomic Content (%)	
	C	O	N
Nitrogen-doped carbon	90.84	1.49	7.67
Nitrogen-free carbon	94.57	5.43	

**Table 2:** Calculated interaction energies (*ΔE*, kCal mol^−1^) for nitrogen-doped porous carbon (NC) and pristine carbon with phosphorus adsorbed on different sites.

Position	O						N			
Functional group	Pyridinic N_COOH	Pyrrolic N_COOH	Nitrogen free_COOH	Pyridinic N_C=O	Pyrrolic N_C=O	Nitrogen free_C=O	Pyridinic N_COOH	Pyrrolic N_COOH	Pyridinic N_C=O	Pyrrolic N_C=O
Interaction energies	−53.619	−20.233	−46.763	−78.462	−84.567	−84.505	−42.251	−11.669	−73.565	−78.061

## Supplementary Materials

The following are available online at www.mdpi.com/1996-1944/11/1/134/s1. Figure S1: (a) SEM images of a RPC composite particle; (b,c) EDS mapping showing distribution of carbon and phosphorus, Figure S2: The optimized geometries of phosphorus adsorbed on different active sites.
