# Supplementary material for: Nitrogen-Doped Carbon for Red Phosphorous Based Anode Materials for Lithium Ion Batteries
Source: Materials (Basel). 2018 Jan 15;11(1):134. doi: 10.3390/ma11010134 (PMC5793632; doi:10.3390/ma11010134)
Supplement: Supplementary file 1 [file materials-11-00134-s001.pdf]

Nitrogen-doped Carbon for Red Phosphorous Based Anode Materials for Lithium Ion Batteries

Jiaoyang Li [1,2], Yumin Qian [3], Li Wang [1,*] and Xiangming He [1,*]

[1] Institute of Nuclear & New Energy Technology, Tsinghua University, Beijing 100084, China; ljyljy1801@126.com
[2] Department of Mechanical Engineering, Massachusetts Institute of Technology, Cambridge, MA 02139, USA
[3] Institute of Functional Nano & Soft Materials, Soochow University, Suzhou 215123, China; yuminqianwk@foxmail.com
[*] Correspondence: wang-l@tsinghua.edu.cn (L.W.); hexm@tsinghua.edu.cn (X.H.); Tel.: +86-10-6279-4226 (X.H.)

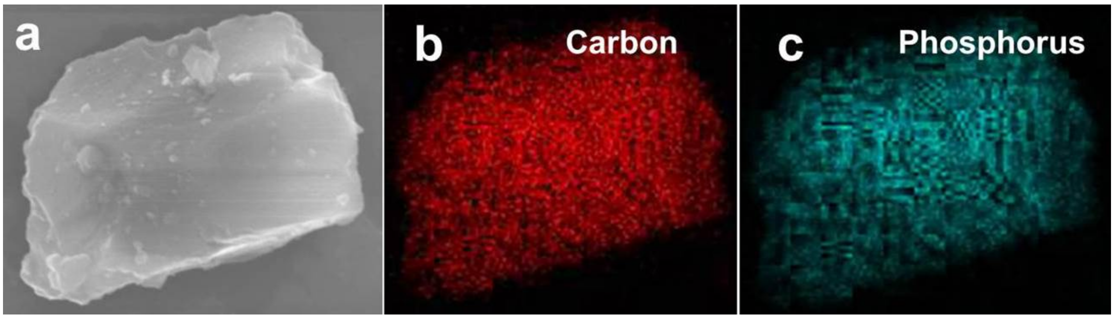

Figure S1. (a) SEM images of a RPC composite particle; (b,c) EDS mapping showing distribution of carbon and phosphorus.

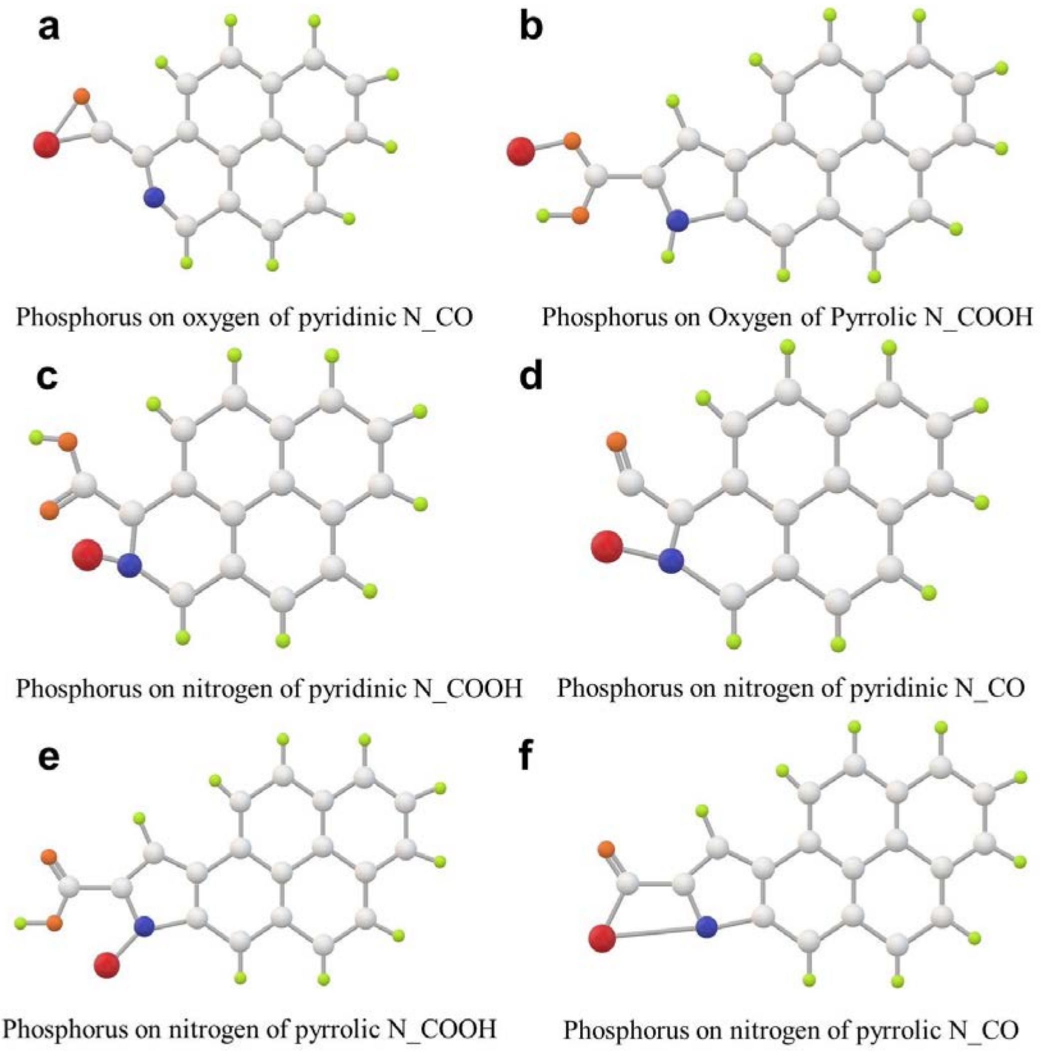

Figure S2. The optimized geometries of phosphorus adsorbed on different active sites.
